# Benzamide oxime

**DOI:** 10.1107/S1600536808020813

**Published:** 2008-07-12

**Authors:** Shu-Qing Xu, Jia-Ming Li

**Affiliations:** a Laboratory of Beibu Gulf Marine Protection and Exploitation, Department of Chemistry and Biology, Qinzhou University, Qinzhou, Guangxi 535000, People’s Republic of China

## Abstract

In the crystal structure of the title compound, C_7_H_8_N_2_O, mol­ecules are connected *via* inter­molecular N—H⋯O and O—H⋯N hydrogen bonds to form a two-dimensional supra­molecular structure. The oxime group has an *E* configuration and the dihedral angle between the mean planes of the benzene ring and the amidoxime grouping is 20.2 (3)°.

## Related literature

For related literature, see: Bruton *et al.* (2003 ▶); Kang *et al.* (2007 ▶); Li *et al.* (2007 ▶); Srivastava *et al.* (1997 ▶); Wang *et al.* (2006 ▶, 2007 ▶); Bertolasi *et al.* (1982 ▶); Chertanova *et al.* (1994 ▶); Goel *et al.* (1981 ▶); Xing, Ding *et al.* (2007 ▶); Xing, Wang *et al.* (2007 ▶).
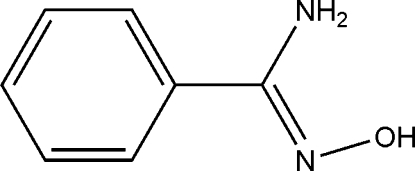


## Experimental

### 

#### Crystal data

C_7_H_8_N_2_O
*M* *_r_* = 136.15
Monoclinic, 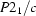

*a* = 12.579 (2) Å
*b* = 5.053 (1) Å
*c* = 10.908 (2) Å
β = 90.380 (7)°
*V* = 693.3 (2) Å^3^
*Z* = 4
Mo *K*α radiation
μ = 0.09 mm^−1^
*T* = 273 (2) K
0.28 × 0.22 × 0.18 mm

#### Data collection

Bruker SMART CCD area-detector diffractometer
Absorption correction: multi-scan (*SADABS*; Sheldrick, 1996 ▶) *T*_min_ = 0.975, *T*_max_ = 0.984
4489 measured reflections
1216 independent reflections
967 reflections with *I* > 2σ(*I*)
*R*_int_ = 0.028

#### Refinement

*R*[*F* ^2^ > 2σ(*F* ^2^)] = 0.050
*wR*(*F* ^2^) = 0.145
*S* = 1.04
1216 reflections
92 parameters
H-atom parameters constrained
Δρ_max_ = 0.21 e Å^−3^
Δρ_min_ = −0.22 e Å^−3^

### 

Data collection: *SMART* (Bruker, 2003 ▶); cell refinement: *SAINT* (Bruker, 2003 ▶); data reduction: *SAINT*; program(s) used to solve structure: *SHELXS97* (Sheldrick, 2008 ▶); program(s) used to refine structure: *SHELXL97* (Sheldrick, 2008 ▶); molecular graphics: *SHELXTL* (Sheldrick, 2008 ▶); software used to prepare material for publication: *SHELXTL*.

## Supplementary Material

Crystal structure: contains datablocks global, I. DOI: 10.1107/S1600536808020813/ez2131sup1.cif

Structure factors: contains datablocks I. DOI: 10.1107/S1600536808020813/ez2131Isup2.hkl

Additional supplementary materials:  crystallographic information; 3D view; checkCIF report

## Figures and Tables

**Table 1:** Hydrogen-bond geometry (Å, °)

*D*—H⋯*A*	*D*—H	H⋯*A*	*D*⋯*A*	*D*—H⋯*A*
O1—H1⋯N2^i^	0.82	2.10	2.820 (2)	147
N1—H1*A*⋯O1^ii^	0.86	2.29	3.031 (2)	145

Symmetry codes: (i) 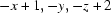; (ii) 
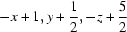
.

**Fig. 1.**
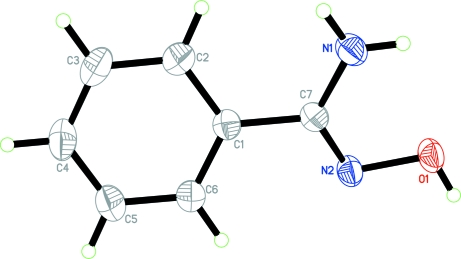
The molecular structure of I showing the atom numbering scheme with displacement ellipsoids at the 30% probability level.

**Fig. 2.**
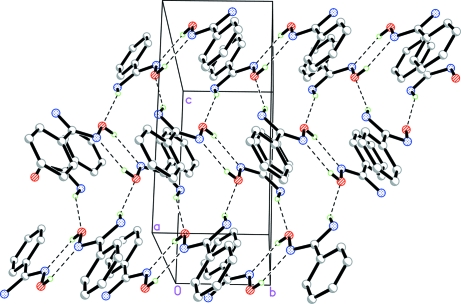
Part of the crystal structure showing hydrogen bonds as dashed lines. H atoms, except for those involved in hydrogen bonds, are not included.

## supplementary crystallographic information

### Comment

The synthesis of Schiff base complexes containing oxime (–C═N—OH) functional groups has attracted great interest due to their antiviral, anticancer and antibacterial activities (Srivastava *et al.*, 1997; Goel *et al.*, 1981; Li *et al.*, 2007; Wang *et al.*, 2007; Xing, Ding *et al.* (2007), Xing, Wang *et al.* (2007). Also, the interesting hydrogen-bond systems in the crystal structures of oximes have been analysed and a correlation between the pattern of hydrogen bonding and N—O bond lengths has been suggested (Bertolasi *et al.*, 1982; Bruton *et al.*, 2003). Herein, we report the synthesis and crystal structure of the title compound, (I). In the crystal structure of the title compound, molecules are connected *via* intermolecular N—H···O and O—H···N hydrogen bonds (see Table 1 and Fig. 2) to form a two-dimensional supramolecular structure. The oxime group has an E configuration [C4—C9—N1—O3 = -179.43 (14) °, Chertanova *et al.*, 1994] and the dihedral angle between the mean planes of the benzene ring and the C7/N1/N2/O grouping is 20.2 (3) °, which is less than that reported for similar structures by Kang *et al.* (2007) and Xing, Ding *et al.* (2007), Xing, Wang *et al.* (2007).

### Experimental

Reagents and solvents used were of commercially available quality. The Schiff base ligand benzamidoxime was synthesized according to the method of Kang *et al.* (2007). A mixture of benzonitrile (0.33 mol) and hydroxylamine hydrochloride (0.33 mol) in ethanol (231 ml) and potassium carbonate (0.33 mol) in water (66 ml) was refluxed for 12 h. After cooling and filtering, compound (I) was obtained. Crystals of (I) suitable for X-ray diffraction were obtained by slow evaporation of an ethanol solution.

### Refinement

H atoms were positioned geometrically, with N—H = 0.86 A (for NH), O—H = 0.82 Å (for OH) and C—H = 0.93 Å for aromatic H atoms, and constrained to ride on their parent atoms, with *U*_iso_(H) = xUeq(C,*N*,*O*), where *x* = 1.5 for OH H, and *x* = 1.2 for all other H atoms.

### Crystal data

**Table:**

C_7_H_8_N_2_O	*F*_000_ = 288
*M**_r_* = 136.15	*D*_x_ = 1.304 Mg m^−^^3^
Monoclinic, *P*2_1_/*c*	Mo *K*α radiation λ = 0.71073 Å
Hall symbol: -P 2ybc	Cell parameters from 1198 reflections
*a* = 12.579 (2) Å	θ = 2.5–27.7º
*b* = 5.053 (1) Å	µ = 0.09 mm^−^^1^
*c* = 10.908 (2) Å	*T* = 273 (2) K
β = 90.380 (7)º	Block, colorless
*V* = 693.3 (2) Å^3^	0.28 × 0.22 × 0.18 mm
*Z* = 4	

### Data collection

**Table:**

Bruker SMART CCD area-detector diffractometer	1216 independent reflections
Radiation source: fine-focus sealed tube	967 reflections with *I* > 2σ(*I*)
Monochromator: graphite	*R*_int_ = 0.028
*T* = 273(2) K	θ_max_ = 25.0º
φ and ω scans	θ_min_ = 1.6º
Absorption correction: multi-scan(SADABS; Sheldrick, 1996)	*h* = −14→14
*T*_min_ = 0.975, *T*_max_ = 0.984	*k* = −6→6
4489 measured reflections	*l* = −12→12

### Refinement

**Table:**

Refinement on *F*^2^	Secondary atom site location: difference Fourier map
Least-squares matrix: full	Hydrogen site location: inferred from neighbouring sites
*R*[*F*^2^ > 2σ(*F*^2^)] = 0.050	H-atom parameters constrained
*wR*(*F*^2^) = 0.145	*w* = 1/[σ^2^(*F*_o_^2^) + (0.0753*P*)^2^ + 0.2452*P*] where *P* = (*F*_o_^2^ + 2*F*_c_^2^)/3
*S* = 1.05	(Δ/σ)_max_ < 0.001
1216 reflections	Δρ_max_ = 0.21 e Å^−^^3^
92 parameters	Δρ_min_ = −0.22 e Å^−^^3^
Primary atom site location: structure-invariant direct methods	Extinction correction: none

### Special details

**Table:**

Geometry. All e.s.d.'s (except the e.s.d. in the dihedral angle between two l.s. planes) are estimated using the full covariance matrix. The cell e.s.d.'s are taken into account individually in the estimation of e.s.d.'s in distances, angles and torsion angles; correlations between e.s.d.'s in cell parameters are only used when they are defined by crystal symmetry. An approximate (isotropic) treatment of cell e.s.d.'s is used for estimating e.s.d.'s involving l.s. planes.
Refinement. Refinement of *F*^2^ against ALL reflections. The weighted *R*-factor *wR* and goodness of fit *S* are based on *F*^2^, conventional *R*-factors *R* are based on *F*, with *F* set to zero for negative *F*^2^. The threshold expression of *F*^2^ > σ(*F*^2^) is used only for calculating *R*-factors(gt) *etc*. and is not relevant to the choice of reflections for refinement. *R*-factors based on *F*^2^ are statistically about twice as large as those based on *F*, and *R*- factors based on ALL data will be even larger.

### Fractional atomic coordinates and isotropic or equivalent isotropic displacement parameters (Å^2^)

**Table:**

	*x*	*y*	*z*	*U*_iso_*/*U*_eq_	
O1	0.50237 (11)	0.1813 (3)	1.12339 (12)	0.0523 (4)	
H1	0.4578	0.0750	1.0990	0.078*	
N2	0.58736 (13)	0.1959 (3)	1.03730 (14)	0.0453 (5)	
C7	0.63885 (14)	0.4152 (4)	1.05147 (16)	0.0391 (5)	
N1	0.61721 (14)	0.5943 (3)	1.13971 (15)	0.0508 (5)	
H1A	0.5666	0.5653	1.1906	0.061*	
H1B	0.6540	0.7373	1.1450	0.061*	
C1	0.72942 (14)	0.4640 (4)	0.96838 (17)	0.0411 (5)	
C5	0.8218 (2)	0.3813 (6)	0.7814 (2)	0.0710 (7)	
H5	0.8256	0.2908	0.7073	0.085*	
C6	0.73762 (19)	0.3377 (5)	0.8576 (2)	0.0643 (7)	
H6	0.6847	0.2195	0.8337	0.077*	
C2	0.8089 (2)	0.6386 (6)	0.9997 (3)	0.0781 (8)	
H2	0.8062	0.7276	1.0742	0.094*	
C4	0.89920 (19)	0.5537 (5)	0.8122 (2)	0.0674 (7)	
H4	0.9560	0.5839	0.7599	0.081*	
C3	0.8926 (2)	0.6829 (7)	0.9215 (3)	0.0962 (11)	
H3	0.9455	0.8027	0.9437	0.115*	

### Atomic displacement parameters (Å^2^)

**Table:**

	*U*^11^	*U*^22^	*U*^33^	*U*^12^	*U*^13^	*U*^23^
O1	0.0545 (9)	0.0537 (9)	0.0488 (8)	−0.0083 (6)	0.0195 (7)	0.0020 (6)
N2	0.0477 (9)	0.0438 (10)	0.0445 (9)	−0.0035 (7)	0.0130 (7)	0.0000 (7)
C7	0.0435 (10)	0.0375 (10)	0.0364 (9)	0.0027 (8)	0.0008 (7)	0.0041 (7)
N1	0.0609 (11)	0.0444 (10)	0.0471 (9)	−0.0028 (8)	0.0116 (8)	−0.0054 (8)
C1	0.0427 (10)	0.0376 (10)	0.0429 (10)	0.0011 (8)	0.0024 (8)	0.0036 (8)
C5	0.0705 (15)	0.0845 (18)	0.0583 (13)	−0.0135 (13)	0.0222 (12)	−0.0124 (13)
C6	0.0606 (13)	0.0763 (16)	0.0562 (13)	−0.0226 (12)	0.0151 (10)	−0.0164 (12)
C2	0.0739 (16)	0.0848 (19)	0.0757 (16)	−0.0334 (14)	0.0208 (13)	−0.0281 (14)
C4	0.0543 (13)	0.0735 (16)	0.0747 (16)	−0.0065 (12)	0.0226 (11)	0.0063 (13)
C3	0.0763 (18)	0.108 (2)	0.105 (2)	−0.0503 (18)	0.0278 (16)	−0.0271 (19)

### Geometric parameters (Å, °)

**Table:**

O1—N2	1.430 (2)	C5—C4	1.348 (4)
O1—H1	0.8200	C5—C6	1.368 (3)
N2—C7	1.292 (2)	C5—H5	0.9300
C7—N1	1.350 (2)	C6—H6	0.9300
C7—C1	1.481 (3)	C2—C3	1.378 (4)
N1—H1A	0.8600	C2—H2	0.9300
N1—H1B	0.8600	C4—C3	1.362 (4)
C1—C6	1.371 (3)	C4—H4	0.9300
C1—C2	1.375 (3)	C3—H3	0.9300
			
N2—O1—H1	109.5	C6—C5—H5	119.6
C7—N2—O1	109.99 (15)	C5—C6—C1	121.6 (2)
N2—C7—N1	123.82 (17)	C5—C6—H6	119.2
N2—C7—C1	117.16 (16)	C1—C6—H6	119.2
N1—C7—C1	118.97 (17)	C1—C2—C3	120.5 (2)
C7—N1—H1A	120.0	C1—C2—H2	119.7
C7—N1—H1B	120.0	C3—C2—H2	119.7
H1A—N1—H1B	120.0	C5—C4—C3	118.7 (2)
C6—C1—C2	117.3 (2)	C5—C4—H4	120.7
C6—C1—C7	121.63 (18)	C3—C4—H4	120.7
C2—C1—C7	121.06 (18)	C4—C3—C2	121.0 (2)
C4—C5—C6	120.9 (2)	C4—C3—H3	119.5
C4—C5—H5	119.6	C2—C3—H3	119.5
			
O1—N2—C7—N1	3.2 (2)	C2—C1—C6—C5	0.5 (4)
O1—N2—C7—C1	−179.43 (14)	C7—C1—C6—C5	−179.5 (2)
N2—C7—C1—C6	21.8 (3)	C6—C1—C2—C3	0.2 (4)
N1—C7—C1—C6	−160.7 (2)	C7—C1—C2—C3	−179.8 (3)
N2—C7—C1—C2	−158.2 (2)	C6—C5—C4—C3	0.5 (5)
N1—C7—C1—C2	19.3 (3)	C5—C4—C3—C2	0.2 (5)
C4—C5—C6—C1	−0.9 (4)	C1—C2—C3—C4	−0.5 (5)

### Hydrogen-bond geometry (Å, °)

**Table:**

*D*—H···*A*	*D*—H	H···*A*	*D*···*A*	*D*—H···*A*
O1—H1···N2^i^	0.82	2.10	2.820 (2)	147
N1—H1A···O1^ii^	0.86	2.29	3.031 (2)	145

Symmetry codes: (i) −*x*+1, −*y*, −*z*+2; (ii) −*x*+1, *y*+1/2, −*z*+5/2.
